# Correction for Antonova et al., “Genomic transfer via membrane vesicle: a strategy of giant phage phiKZ for early infection”

**DOI:** 10.1128/jvi.02223-24

**Published:** 2025-01-22

**Authors:** Daria Antonova, Anna Nichiporenko, Mariia Sobinina, Yueqi Wang, Innokentii E. Vishnyakov, Andrey Moiseenko, Inna Kurdyumova, Yuri M. Chesnokov, Elizaveta Stepanchikova, Maria Bourkaltseva, Valeriya R. Samygina, Mikhail Khodorkovskii, Olga S. Sokolova, Maria V. Yakunina

## AUTHOR CORRECTION

Volume 98, no. 10, e00205-24, 2024, https://doi.org/10.1128/jvi.00205-24. Page 13: Acknowledgments, line 12, “RSF (24-44-02003)” should read “Shenzhen First Class Discipline Foundation (to O.S.S.).”

Page 13: Funding table, “Russian Science Foundation” should read “Shenzhen First Class Discipline Foundation.” Grant number 24-44-02003 should be removed.

